# An Investigation of Parturient Ocular Appearance in Sows

**DOI:** 10.3390/ani14182693

**Published:** 2024-09-16

**Authors:** Alexandra Walls, Evelyn Hall, Sabrina Lomax, Roslyn Bathgate

**Affiliations:** 1 Sydney School of Veterinary Science, Faculty of Science, The University of Sydney, Sydney 2006, Australia; 2 School of Life and Environmental Sciences, Faculty of Science, The University of Sydney, Sydney 2006, Australia

**Keywords:** eye redness, farrowing, pig, abnormal parturition, straining

## Abstract

**Simple Summary:**

Sow parturition, the process of giving birth to piglets, is a highly stressful process, with outcomes affecting farm economics as well as sow and piglet welfare. Protocols that assist with the identification of abnormal parturition are required for optimal production outcomes. The eye is an easily accessible and anatomically consistent body region, often used to aid medical diagnoses. A novel three-level ocular scoring system was devised and used to investigate the relationship between changes in ocular appearance and farrowing kinetics. It was determined that ocular appearance scoring can be used at farrowing to aid identification of sows experiencing an abnormal parturition, as changes are associated with increased straining and extended farrowing durations. This is a first step in developing a scale that may aid in predicting an extended parturition (>300 min), identifying sows that require assistance and so aiding staff management, sow welfare, and piglet survival.

**Abstract:**

Protocols that enable prompt identification of sows in need of assistance during farrowing are important for optimal production outcomes. Change in the colour and appearance of the sclera of the eye can indicate increased stress. This warrants investigation into its use for the identification of sows in need of assistance at the time of parturition. To this end, a three-level ocular scoring system for the pig was devised and subsequently used in a preliminary investigation into the relationship between farrowing kinetics and visual changes in the sclera at farrowing. Data were collected and analysed from twenty randomly selected Large White × Landrace crossbred sows during farrowing. It was determined that sows with a severe ocular score were more likely to experience a prolonged farrowing duration (*p* = 0.013) and incur increased parturient straining of either total straining time (*p* = 0.011) or straining per piglet (*p* = 0.025). There was a significant association between ocular score and litter size (*p* = 0.043). Ocular score was not associated with sow parity (*p* = 0.728) and inter-piglet interval (*p* = 0.075). The proposed three-level scoring system successfully identified sows experiencing an abnormal parturition as defined by a prolonged farrowing duration and increased straining time. Findings from this study suggest the potential application of this simple ocular scoring tool for identifying sows experiencing an abnormal farrowing via in-person application or integration into remote monitoring systems in the future.

## 1. Introduction

Parturition is a complex, multi-factorial process requiring substantial physiological expenditure, particularly for polytocous species such as the sow [1]. For the well-being of sows and their piglets, smooth and successful transitions throughout the parturient phases of dilation, foetal birth, and placental expulsion are essential. Sow factors such as parity (the number of previous farrowings), litter size, body condition score, muscular strength, contraction intensity, and individual anatomical makeup can affect parturition outcome [2,3,4]. Additionally, piglet factors, including foetuses with reduced vitality and foetal positioning, pose additional challenges to the success of parturition [2,4]. With so many confounding parturient factors, abnormal parturition (dystocia) is thought to affect up to 47% of spontaneous farrowings [5]. Within industry, time measurement tools such as inter-piglet intervals greater than 30–45 min [5,6,7] or farrowing durations exceeding 300 min [8] are used to identify animals with increased risk of poor parturient outcome. However, the effectiveness of these tools is limited to staff time and the ability to monitor continuously. A novel dystocia identification tool, requiring a less labour-intensive application, is required within the pork industry, which is affected by labour shortages [9].

Secretion of maternal cortisol before and during parturition is a necessary endocrine change for the initiation and completion of farrowing [10]. Additional increases in cortisol above elevated prepartum baselines indicate increased parturient stress [10]. Simple visual identifiers of increased stress could aid in defining dystocia in sow parturition for identification and treatment of animals requiring assistance. The eye is an easily accessible, anatomically consistent organ that can aid diagnosis of medical conditions that are not specific to the eye region [11]. For example, in humans, yellowing of the sclera is linked to decreased liver function [12] and inflammation of the sclera can flag autoimmune conditions like rheumatoid arthritis [13]. Hence, identifying a connection between parturient stress caused by an abnormal parturition and ocular appearance could assist decision-making at farrowing for improved sow and piglet welfare.

Recognising pain and/or stress in sows can be particularly challenging due to their innate nature as a prey species to conceal signs of vulnerability [14]. Understanding this becomes important when identifying increased stress at parturition, a time when a degree of stress is expected [3]. For this reason, previous research has focused on developing non-invasive behavioural tools that can aid in identifying sows with increased pain [3,15,16]. Behaviours including tail flicking, pawing, legging, and facial grimacing have been used to successfully detect parturient stress [15,16]. However, accurate identification of behavioural indicators like these takes time to master and requires extensive training and experience of observers. Consequently, the need for simple observational tools with minimal room for bias are necessary for early identification of increased parturient stress.

Utilising the eye and surrounding tissue to assess parturient pain in sows via grimace scaling has been an integral step towards identifying pain at parturition within the sow [15]. Yet, there is a notable absence of studies exploring the relationship between ocular appearance and the parturient experience. In times of heightened stress, hypertension can be caused by stimulation of the nervous system to produce vasoconstricting hormones and increased blood flow [17]. Hypertension can cause the sclera of the eye to appear red as blood vessels within the eye constrict [18]. A visual indication of stress, such as a change in sclera colour, could aid in prompt decision-making at parturition when early identification of animals needing assistance can affect both sow and piglet survival.

With the absence of simple and immediate indicators of heightened stress during parturition within the sow, novel tools are necessary to identify animals requiring assistance. Consequently, this study investigated the relationship between ocular appearance and farrowing kinetics as an initial step towards creating a tool for identifying sows with increased parturient stress.

## 2. Materials and Methods

This study was conducted at The University of Sydney commercial piggery in Camden, NSW, between 2022 and 2023. Permission to undertake the study was granted by The University of Sydney Animal Ethics Committee (2023/0481).

### 2.1. Animals, Management, and Housing

Data were collected from twenty randomly selected Large White × Landrace sows. Sows were moved from group housing to individual Jyden Sow Welfare and Piglet Protection pens (SWAP) 5–7 days before expected farrowing dates, calculated as 114 days from insemination. Sows were confined by moveable swing-side crates 4–5 days before expected due dates and for 7–10 days following farrowing. Ambient shed temperature was maintained at 22 ± 2 °C. Sows were provided with two feedings of commercial grower pellets totalling 3 kgs daily at 07:00 and 15:00. Sows were supplied with water ad libitum through nipple drinkers at the front of each crate. Nesting material in the form of wood shavings and straw was accessible ad libitum to each animal via mounted straw racks. Only sows with a body condition score between 2.5–3.5 were considered for this study. All sows were naturally mated and experienced an unassisted farrowing.

### 2.2. Data Collection

Each morning, from three days before the expected farrowing date, a photograph of each sow’s eye was taken using an iPhone 13 (Apple Inc., Cupertino, CA, USA) with a KEROST portable LED ring light (Kerost, Australia, https://kerost.com/) attached. The ring light was set to white light to create consistency between photos where ambient light varied throughout the room. Photos were captured from an approximate distance of 30 cm, making sure to include the whole eye within each image. Where possible, the same eye was used for all photos of each sow. Sow positioning did not affect image capturing providing the whole eye was accessible. Each photo was labelled with the sow identification number, parity, the date, and number of piglets born before the photo was taken.

At the commencement of the expulsion phase of parturition, as defined by the birth of the first piglet, an additional ocular photo was taken following the same guide as previously mentioned. For every following piglet, an ocular photo was taken immediately after birth. Farrowing was deemed completed after the expulsion of the placentae, and no further images were collected.

Throughout parturition, each sow pen was continuously monitored via fitted fixed Vari-Focal Dome Cameras (VIP Vision Professional AI 4 MP 2.7–13.5 mm, Cornick Pty Ltd., Melbourne, Australia) running alongside a Watchguard Compact Network 16 Channel Video Recorder with PoE (Watchguard, Seattle, WA, USA). Post-farrowing, video files for each parturition were extracted from 1 h before parturition until 1 h after placental expulsion. Extracted video files were labelled with sow identification number, parity, and farrowing date. Video data were visually analysed twice by two different individuals, starting from 1 h before the birth of the first piglet until immediately after the last piglet was born. Data were later averaged between the two data collectors before the start of data analysis to minimise the effects of human bias.

Video data were reviewed at normal speed and the duration of abdominal straining behaviour was recorded for each piglet (defined as the total sum of straining behaviour occurring in the time immediately after the birth of one piglet until immediately prior to the next). Abdominal straining for the first piglet born in each litter was acquired by examining video data from one hour before birth and accumulating the sum of all straining behaviour within this time. Abdominal strain was defined as visible abdominal contraction occurring between periods of abdominal relaxation. The behaviour was often but not always presented with fore and hind legs tightly pressed to the abdomen and forehead brought down towards the chest. In the event of uncertainty, videos were rewound and re-examined. Often, straining encompassed several small abdominal contractions. If no clear break occurred between the start of one contraction and the next, these events were recorded as one event. Additionally, farrowing duration (defined as the time between the birth of the first piglet and the last), litter size, inter-piglet intervals (defined as the minutes between the birth of one piglet and the next) were recorded.

### 2.3. Formulation of Ocular Grading Scale and Sow Assignment

**Figure 1:**
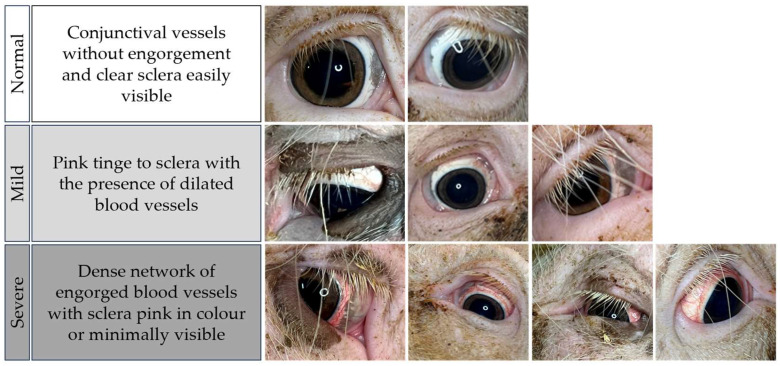
A three-level scoring system with photographs depicting varying levels of ocular appearance in the sow at parturition.

A grading scale was created by adapting and simplifying previously developed scales used in humans [19,20]. Based on previously observed ocular appearances and indicators of sow stress in preliminary studies (unpublished data), three tiers (normal, mild, and severe) were considered optimal for acceptance and integration into use by industry. Ocular appearance criteria focused on differentiating levels of blood vessel presence and appearance as well as sclera colour and visibility (Figure 1).

To determine ocular scores for each sow, individual ocular images were assigned either “normal”, “mild”, or “severe” using the descriptions outlined in Figure 1. The highest score attained across each parturition was used to determine final ocular appearance score for that sow.

### 2.4. Data Preparation and Calculations

Parity was categorised into two groups, either “primiparous” (parity 0) or “multiparous” (parity 1 and above). Total straining for each sow was calculated as the total duration of abdominal straining over the course of observation. The average total straining per piglet for each sow was calculated by dividing the total straining time by litter size. The average inter-piglet interval for each sow was calculated by dividing the sum of the inter-piglet interval for each litter by litter size. Maximum strain (the period in which a sow strained the longest for a piglet out of her litter) was calculated by identifying the highest abdominal straining score acquired by a piglet in each litter in minutes.

### 2.5. Statistical Analysis

Data analysis was conducted using RStudio^®^ (v1.4.1564) for R (v4.0.2) [21] utilising the packages lme4, emmeans, ordinal, and RVAideMemoire. Data were expressed as mean ± standard error (SE) for continuous outcome variables and for all analyses, a *p* value of <0.05 was considered statistically significant. The effect of ocular score on each farrowing variable (farrowing duration, total straining, average total straining per piglet, average inter-piglet interval, maximum strain, and litter size) was assessed using univariable linear modelling (LM). Post-hoc multiple comparison tests (emmeans) with Tukey adjustment were performed on all farrowing variables against ocular score to determine pairwise differences. An ordinal logistic regression model (clm) was used to assess the association between ocular score and parity, with ocular score as the outcome.

## 3. Results

Sows (*n* = 20) were of parity 1.1 ± 0.29 (mean ± SE, range 0–7) and gave birth to 12 ± 0.51 (range 5–17) piglets, with 11.15 ± 0.52 (range 4–17) liveborn and 0.85 ± 0.13 (range 0–2) stillborn.

All sows were recorded as having a ‘normal’ ocular score in the photos taken prior to farrowing commencing. In photos taken during parturition, the majority of sows (40%, *n* = 8) experienced a severe ocular score, followed by normal (35%, *n* = 7) and mild (25%, *n*= 5) scores, respectively. Results from linear models and pairwise comparisons are summarised in Table 1.

### 3.1. Association between Ocular Score and Farrowing Duration

Sows with a severe ocular score had a significantly longer farrowing duration compared to sows with a normal or mild score (*p* = 0.013; Table 1).

### 3.2. Association between Ocular Score and Straining

Sows with a severe ocular score had significantly longer total straining times than sows with a normal or mild score (*p* = 0.011; Table 1). Sows with a severe ocular score had significantly prolonged average total straining per piglet time than those with a normal score (*p* = 0.025; Table 1). There was no statistically significant difference in average total straining per piglet with animals that received a mild score and those with a normal or severe score (*p* ≥ 0.146). Sows with a severe ocular score displayed significantly longer maximum strain times than sows with a normal or mild score (*p* = 0.004; Table 1).

### 3.3. Association between Ocular Score and Inter-Piglet Intervals

No statistically significant relationship was observed between average inter-piglet interval and ocular score (*p* = 0.075; Table 1).

### 3.4. Association between Ocular Score and Litter Size

There was a significant association between litter size and ocular score where mild score cases had a lower average litter size than normal score cases (*p* = 0.043; Table 1), with no difference seen between severe and mild score cases.

### 3.5. Association between Parity and Ocular Score

No statistically significant relationship was observed between ocular scores of multiparous and primiparous sows at farrowing (*p* = 0.728).

## 4. Discussion

This is the first time that an association between parturient experience and ocular features in sows has been reported. Additionally, the present study was the first to explore ocular scoring as a potential tool for identifying sows experiencing increased stress during parturition with the aim of improving both sow and piglet welfare. Most significantly, sows with a severe ocular score were more likely to experience a prolonged farrowing duration, increased total straining time or increased straining time for a piglet, all of which have been identified in the literature as being associated with poor parturient outcome [1,8,22,23,24].

Dystocia within the sow is commonly defined as a parturition in which a prolonged duration occurs either between piglets [5] or over the total farrowing [8]. A total farrowing duration exceeding 300 min is associated with having a higher repeat breeding rate in subsequent cycles [25], suggestive of damage to the reproductive tract and a trait undesirable for attaining profitability with a commercial system. The current study had a predicted mean farrowing duration for sows with a severe ocular score of 354 min compared with 213 and 206 min for sows with a normal and mild ocular score, respectively. This supports the >300 min rule [8]. Further investigation with a larger population size is necessary to confirm the potential for ocular scoring to pre-emptively flag extended farrowing durations before they occur. Early flagging of a prolonged farrowing could reduce associated adverse effects such as piglet mortality and decreased subsequent sow fertility if this information is promptly and appropriately acted upon.

The prevalence of dystocia within the modern pork production system is contested, with reported rates ranging from 1% to 47% of spontaneous farrowings [1,5,6]. The definition of dystocia in the sow at parturition is another point of contention, but it undoubtedly includes the failure to expel foetuses [23]. At what length of time or with what other signal it is appropriate to intervene is a complex decision and may not be as simple as using one basic tool. If the definition of dystocia in sow parturition included “difficulty expelling foetuses”, then evaluation of abdominal straining could provide a clearer indication of increased stress than time-based identification tools that do not account for physiological needs at farrowing. Findings from this study demonstrate increased abdominal straining in sows experiencing an abnormal parturition, with a period of straining exceeding 10.36 min more likely to incur a severe ocular score. Further work is required to determine if this is indictive of hypertension as a result of difficulties during parturition.

Non-significant interactions between longer inter-piglet intervals and ocular scores pose contradictory evidence for the use of inter-piglet time-based tools used within modern pork production. In an attempt to reduce piglet losses, some systems advocate for manual obstetrical intervention with inter-piglet intervals as small as 30 min [7]. Industry recommendation is that approximately 10% of farrowings require intervention, but in practice manual farrowing assistance occurs in up to 60% of spontaneous farrowings [7,26]. The contrast between acceptable intervention rates and actual occurrences within the intensive pork industry implies that current practices are either unsuitable or, at the very least, warrant re-evaluation.

Inter-piglet time-based tools fail to account for a production system where litter sizes have grown from an average of 11.5 piglets in 1966 to 14 piglets in 2019 [23]. With the increase in the physiological demands of expelling a larger litter size, it is likely that periods of rest are needed throughout the process, which is also observed in whelping dogs [27]. Time-related tools of modern intensive pig production fail to consider necessary rest periods in farrowing and potentially risk the health of sows due to possible uterine tearing or metritis associated with unnecessary obstetrical removal of piglets [26]. Although time-related dystocia tools are used to improve piglet survival, it is done at the potential detriment of future sow reproductivity [7] and economic success of production. Application of tools like the proposed ocular scoring system could help discriminate sows experiencing a pause in farrowing and those experiencing increased parturient stress for a reduction in potentially detrimental obstetrical intervention procedures.

As the average intensive pig herd size increases to maintain profitability, each staff member is required to care for a larger number of animals. This reduces the time and attention paid to individual animals, even in crucial times such as parturition, where it has been shown that observation is vital for piglet survival [28]. The proposed ocular scoring system could aid alongside already established procedures with sows experiencing a severe score warranting further management. Although the proposed tool still requires time to apply in its infancy, it does not require the continuous monitoring that inter-piglet interval tools involve. Additionally, with the progression of technology within intensive systems, ocular scoring has the potential to be adapted to video/sensor-based technologies, like the success seen in preliminary remote pig-weight machine learning technologies, which reduces human–pig contact and measurement errors [29]. Additionally, integrating early dystocia detection tools such as the proposed ocular scoring system within pork production systems allows for discrimination between sows undergoing normal parturition and those experiencing abnormal parturition that warrants intervention. A decrease in unnecessary manual intervention could reduce subsequent sow wastage and improve production profitability.

The present study did not investigate piglet outcome and so cannot comment on whether intervention was warranted for piglet survival in any of the included farrowings. In some cases, intervention may have occurred on properties with obstetric protocols in place. Although all sows from the present study successfully expelled piglets without assistance, 40% experienced a severe ocular score. This poses the question: do all cases of dystocia warrant intervention? The present study, by the previously described definition of dystocia (farrowing > 300 min), had 20% of sows experiencing dystocia. All sows within the present study that farrowed with a duration > 300 min had a severe ocular score, but not all sows with a severe score experienced a farrowing duration > 300 min. Additionally, no intervention was undertaken in any of the observed farrowings and thus only with further research can an answer to the proposed question be given. However, it is clear that ocular scores could aid in decision-making where extra attention is given to sows with higher scores. Additionally, sows that experience a severe score should be monitored for future occurrence in subsequent farrowings. Continual occurrence of severe scores may be undesirable within herds and could aid as an additional selection criterion for gilt retention or sow culling. Further investigation within a larger population is necessary to determine the appropriate application of ocular scores to management plans for farrowing sows.

Anatomical facial structure and breed characteristics could hinder the usability of ocular scoring in some production settings. Obtaining a visual on ocular appearance could be problematic in older parity or higher body condition-scored sows with additional skin around the eye, as excess skin can minimise the visibility of the sclera. Additionally, breeds such as Berkshire or Meishan, which inherently carry significant excess skin around the face, could encounter similar issues. These issues were not experienced in the present study, which included only sows of predominately Landrace x Large White bloodlines without excessive facial skin. With similar breeds making up the large majority of pork production in Australia [30], and internationally [31], the likelihood of such issues affecting blanket usability within intensive systems would be rare.

## 5. Conclusions

Ocular scoring at parturition may be used to indicate increased stress associated with prolonged straining and parturient duration, parameters indicative of dystocia. Results showed that sows experiencing an extended farrowing duration and a higher incidence of straining were more likely to have a severe ocular score during farrowing. Application of the proposed scoring system within current management protocols could aid in discriminatory decision-making regarding intervention at farrowing. Further investigation with a larger population size is warranted to determine if ocular scores have the ability to pre-emptively flag farrowings > 300 min before they occur and to confirm the effects of litter size on ocular scores observed in this study. Future studies should focus on analysing the association between ocular scores and stillbirth occurrence and piglet survival rates within a piglet’s most vulnerable life stage (within 24 h of birth). Additionally, further studies should investigate other physiological or endocrinological measures of stress, like cortisol levels, alongside ocular scoring to finetune its ability to accurately measure increased stress at parturition. Moreover, future research should determine the usefulness of ocular scoring as an additional selection criterion for gilt retention and sow wastage. Finally, further research is required to determine suitability of ocular scoring for defining an appropriate time for intervention in sows experiencing dystocia for the improved welfare of both sows and piglets at farrowing.

## Figures and Tables

**Table 1:** Summary statistics of univariable linear models (LMs) for the association between ocular score and farrowing kinetics in sows at parturition (*n* = 20). Results are presented as means ± standard error of the mean (SE). Letter superscript indicates the statistically significant differences between ocular scorings within the variable. * *p* < 0.05. The R-squared (R^2^) value signifies the proportion of ocular score explained by the model.

Variable		Predicted mean ± SE	*p*-Value	R^2^
Farrowing duration (min)				
	Normal	213 ± 35.1 ^a^	0.013 *	0.332
	Mild	206 ± 41.5 ^a^		
	Severe	354 ± 32.8 ^b^		
Total straining (min)				
	Normal	15.6 ± 3.62 ^a^	0.011 *	0.344
	Mild	14 ± 4.29 ^a^		
	Severe	30 ± 3.39 ^b^		
Average total straining per piglet (min)				
	Normal	1.42 ± 0.35 ^a^	0.025 *	0.275
	Mild	1.78 ± 0.42 ^ab^		
	Severe	2.83 ±0.33 ^b^		
Maximum strain (min)				
	Normal	4.07 ± 1.3 ^a^	0.004 *	0.408
	Mild	4.52 ± 1.54 ^a^		
	Severe	10.36 ± 1.22 ^b^		
Average inter-piglet interval (min)				
	Normal	15.7 ± 4.68 ^a^	0.075	0.177
	Mild	24.9 ± 5.53 ^a^		
	Severe	31.5 ± 4.37 ^a^		
Litter size (count)				
	Normal	13.6 ± 1.04 ^a^	0.043 *	0.229
	Mild	9.2 ± 1.23 ^b^		
	Severe	12.4 ± 0.97 ^ab^		

## Data Availability

The raw data supporting the conclusions of this article will be made available by the authors on request.
